# History of a Case, in Which the Dura Mater Was Successfully Punctured under the Fontanel

**Published:** 1802-12-01

**Authors:** T. Chevalier

**Affiliations:** South Audley Street


### *History of a Case, in which the Dura Mater was successfully punctured under the Fontanel; communicated by* MR. T. CHEVALIER, *of South Audley Street.*

SEPT. 22, 1802. The daughter of Mr. R. publican, in Oxford Street, a child of a year and an half old, fell out of the ſervant's arms, at ten o'clock in the forenoon, and ſtruck its head violently againſt the edge of a door. It was taken up ſenſeleſs, and Mr. Davis, an Apothecary in Duke Street, was ſent fent for. He immediately applied four leeches to the temples, and gave it a grain of calomel, and a dofe of a cathartic mixture with fenna, which was afterward repeated. The child, notwithftanding, continued in a comatofe ftate for an hour and a half, and then went into convulfions, which had lafted near two hours without any intermiffion, when I was defired to fee her. There was no external mark of injury whatever on the fcalp; but on attentively examining the head, I thought the fontanel appeared fomewhat rounded, and elevated above its natural plane; and from this circumftance, added to the manner in which the fymptoms had advanced, I concluded fome blood-veffel had ruptured underneath the dura mater, and was ftill continuing to bleed. I therefore propofed, as it appeared to be the only chance of faving the child, to expofe the dura mater at the fontanel, and to puncture it, if my fufpicion proved founded. The parents confented to whatever I thought neceffary. While I went home for fome inftruments, I directed the child to be put into a warm bath, and two drops of tinct. opii to be given it. But finding on my return, that no relief had been gained, and the child appearing almoft exhaufted, I performed the operation immediately.

I raifed the fcalp from the whole of the fontanel by a crucial incifion, and then made an angular incifion through the fontanel itfelf; in the angle between the frontal and parietal bones on the right fide, and as near to the parietal bone as could allow me to have fufficient room. I did this on the *right* fide, from obferving that the limbs on the *left* fide were much more violently convulfed than thofe on the right; the contrary fide of the body being ufually moft affected by difeafes exifting on one fide of the brain. On carefully diffecting up the angular incifion which I had made in the fontanel, I was pleafed to fee the blood very diftinctly fhining through the dura mater as far as it became expofed; I therefore cautioufly punctured it with the point of a lancet, and the blood immediately iffued out with confiderable force, fpouting at firft to the diftance of a foot. The continuance of the ftream made me for an inftant fufpect I muft have punctured the longitudinal finus, although I was confident I had expofed too much of the dura mater to run any rifk of that. However, I introduced a bent probe through the puncture, which paffed laterally for fome inches betwixt the dura mater and pia mater under the parietal bone, without the fmalleft refiftance. The blood continued to flow, gradually abating in its force, till between three and four ounces at leaft were difcharged. It appeared venal, but flowed *per saltum*, following the pulfations of the brain. The convulfions were now much lefs violent, and the fontanel was flat on both both fides. The child becoming very faint; a little wine and water with one drop of tinct. opii was given to it, and as the bleeding had ceafed, I dreffed the wound, lying down as much of the fcalp as was not immediately over the puncture in the dura mater, which I thought it beft to keep eafy of accefs for the prefent. She was now put to bed. Her pulfe foon became diftinct, and tolerably regular. The fpafms continued in a very flight degree only, and were gradually abating; they ceafed entirely in three quarters of an hour, when fhe fell afleep. The cathartic mixture was ordered to be repeated every three hours, and a purging glyfter to be given early in the evening.

At ten o'clock in the evening, I found her afleep, but fhe opened her eyes when touched, and the pupil contracted on expofure to light. Her refpiration was eafy and natural; her pulfe was gaining ftrength, and her fkin was fomewhat hot. Two ftools had been procured. She moved both legs and the right arm perfectly well, but not the left arm. The wound therefore was not difturbed. A faline draught with fome infufion of fenna was ordered to be given every four hours.

She paffed a good night, was perfectly fenfible the following morning, and moved both her arms well. The wound was covered with white cerate on lint. In the courfe of the day fhe had feven ftools. She complained much of thirft, and was fick twice from drinking too eagerly. The medicine was ordered to be continued without the fenna.

On the third day, as things were going on in the moft favourable manner, the ficknefs not having returned, the pupils contracting properly, and the child being perfectly fenfible, I laid down the remaining part of the fcalp, and dreffed the wound only fuperficially. From this time nothing particular occurred. She continued lively and well, and on the 20th of October, the fore was entirely healed.

Calling to fee her on the 5th of November, I found her going through the Small-pox, but with very little indifpofition. The eruption was flight, and had not been preceded by any convulfion.

*November* 11, 1802.

